# Use of the Prostate-Specific Antigen Test Among Men Aged 75 Years or Older in the United States: 2006 Behavioral Risk Factor Surveillance System

**Published:** 2010-06-15

**Authors:** Jun Li, Guixiang Zhao, Lori A. Pollack, Judith Lee Smith, Djenaba A. Joseph

**Affiliations:** Centers for Disease Control and Prevention; Centers for Disease Control and Prevention, Atlanta, Georgia; Centers for Disease Control and Prevention, Atlanta, Georgia; Centers for Disease Control and Prevention, Atlanta, Georgia; Centers for Disease Control and Prevention, Atlanta, Georgia

## Abstract

**Introduction:**

In 2008, the US Preventive Services Task Force (USPSTF) updated prostate cancer screening guidelines to recommend against screening for prostate cancer in men aged 75 years or older. We describe the prevalence of prostate-specific antigen (PSA) testing in this population and identify factors that may be correlated with the use of this test.

**Methods:**

Data came from the 2006 Behavioral Risk Factor Surveillance System. We assessed the status of PSA testing in the past year among 9,033 US men aged 76 or older who had no history of prostate cancer. We conducted descriptive and multiple logistic regression analyses to assess associations of PSA testing with certain sociodemographic and psychosocial factors.

**Results:**

Overall, 60% of men aged 76 or older reported having a PSA test in the past year. Men who had health insurance, were satisfied with life, or always had emotional support were significantly more likely to report having a PSA test in the past year. However, men who had no routine health checkup; were divorced, widowed, or separated; or had less than a high school education were significantly less likely to report having had a PSA test.

**Conclusion:**

PSA testing is common among men aged 75 or older in the United States. Certain sociodemographic and psychosocial factors were associated with receipt of this test. This study may not only provide baseline data to evaluate acceptance and implementation of the USPSTF screening guidelines but may also help physicians and public health providers better understand these sociodemographic and psychosocial factors in this population.

## Introduction

Prostate cancer is the most commonly diagnosed nonskin cancer and the second leading cause of cancer death among US men (1). Men with indolent prostate cancers may not experience clinical symptoms during their lifetime; however, these indolent cancers can be detected by a prostate-specific antigen (PSA) screening test. Screening may facilitate curative treatment for prostate cancer but may also lead to overtreatment (2,3). Prostate cancer treatments can cause moderate to substantial harm, including erectile dysfunction, urinary incontinence, bowel dysfunction, and death (4).

In 2008, US Preventive Services Task Force (USPSTF) recommended against screening for prostate cancer in men aged 75 years or older, concluding that the potential harms outweighed the potential benefits (5). The use of the PSA test might be associated with certain sociodemographic and behavioral factors (marital status, education, access to health care, and smoking status) and chronic disease (6-9); however, few studies focused on men aged 75 or older (10). To our knowledge, associations of PSA testing with psychosocial factors such as life satisfaction and emotional support have not been well reported. In this study, we describe the use of PSA testing among US men aged 75 or older and identify potential associations between PSA testing and sociodemographic, psychosocial, and behavioral factors, access to health care, and presence of chronic diseases. The results of this study can establish baseline data to evaluate acceptance and future implementation of new USPSTF prostate cancer screening guidelines. Additionally, this study summarizes potential sociodemographic, psychosocial, and health factors associated with PSA testing in this population.

## Methods

### Data source

Data for this analysis came from the 2006 Behavioral Risk Factor Surveillance System (BRFSS), an ongoing, state-based, random-digit-dialed telephone survey of the noninstitutionalized US civilian population aged 18 years or older. BRFSS collects information on diseases, health-related behaviors, preventive health practices, and access to health care in the United States, including information on prostate cancer testing every 2 years. The 2006 BRFSS data, which contained information on prostate cancer testing, were published immediately before the release of the updated 2008 USPSTF screening guidelines. The median cooperation rate (the percentage of eligible respondents who completed the survey) of the 2006 BRFSS was 75%.

### Study participants and variables

For respondents aged 40 or older, the use of a PSA test within the year before the survey was assessed with 2 questions, and prostate cancer status was determined with a third question (Appendix). In 2006, a total of 101,856 men aged 40 or older completed the BRFSS. Of these, 7,849 men were excluded from analysis because of a history of prostate cancer or missing information on prostate cancer status. We further excluded men younger than 76 (n = 84,228) and men (n = 746) who responded "don't know/not sure" or "refused" or were missing data for the question on prostate cancer screening. The final sample for analysis included 9,033 men aged 76 years or older, representing an estimated population of 4.5 million US men.

In this study, PSA testing in the past year was the primary outcome. To properly categorize men who had the PSA test at age 75 years or older, we used 76 years (at the time of interview) as the cutpoint for age because the survey asked whether the test occurred in the past 12 months. We categorized age into 2 groups in this study: 76 to 80 and 81 or older. We classified race/ethnicity as non-Hispanic white, non-Hispanic black, non-Hispanic Asian/Pacific Islander, Hispanic, and non-Hispanic other race. All respondents who reported that they were of Hispanic/Latino origin were coded as Hispanic. We designated marital status in 3 categories: married/living with a partner, divorced/widowed/separated, or never married. We classified educational status as less than a high school diploma, high school diploma, or more than a high school diploma. We defined smoking status as current smoker (respondents who reported having smoked at least 100 cigarettes during their lifetime and now smoke every day or some days), former smoker (respondents who had smoked at least 100 cigarettes during their lifetime and now do not smoke), or nonsmoker (respondents who have not smoked at least 100 cigarettes in their lifetime). We reclassified levels of emotional support as always (always or usually), sometimes, or rarely/none and reclassified levels of life satisfaction as satisfied (very satisfied or satisfied) or dissatisfied (dissatisfied or very dissatisfied).

We regrouped routine checkup status into a dichotomous variable: had a checkup in the past year or no checkup in the past year. Other dichotomous variables included in this analysis were health insurance, concern about medical cost, veteran status, and diagnosis of diabetes, heart attack, coronary heart disease, or stroke. Respondents who refused to answer, had a missing answer, or did not know the answer to a question were excluded from analysis of the specific question. Missing responses for each variable ranged from 1% to 8%.

### Statistical analyses

The analysis was performed by using SAS-callable SUDAAN version 9.0 (RTI International, Research Triangle Park, North Carolina) to account for the multistage and disproportionate stratified-sampling design. We estimated the weighted percentages and the corresponding 95% confidence intervals (CIs) of men aged 76 or older who reported having (or not having) had a PSA test in the year before the survey by sociodemographic, behavioral, and psychosocial factors and by status of health care access and chronic diseases. Significant differences in the distribution of these factors were determined by χ^2^ test with a *P* value <.05. We conducted a multiple logistic regression analysis to assess relationships between the use of PSA testing and selected variables, adjusting for potential confounders. The full model included an outcome variable (had a PSA test in the past year) and 15 predicted variables: age, race, marital status, education, health insurance, concern about medical cost, routine checkup, veteran status, smoking status, emotional support, life satisfaction, diabetes, heart attack, coronary heart disease, and stroke. The Hosmer-Lemeshow goodness-of-fit test indicated that this model was suitable (*P* = 0.93).

## Results

Among US men aged 76 or older, 60% reported having had a PSA test in the past year (Table 1). The use of a PSA test was significantly higher 1) among men aged 76 to 80 compared with men aged 81 or older; 2) among men married or living with a partner compared with men divorced, widowed, or separated or never married; 3) among men with more than a high school diploma compared with men with a high school diploma or less; and 4) among men who were former smokers compared with men who were nonsmokers (Table 2). Similarly, having health insurance, having had a routine checkup in the past year, being a veteran, being satisfied with life, having had a stroke, and always having emotional support were significantly associated with an increased use of PSA testing.

The multiple logistic regression analysis showed having health insurance, having had a routine checkup, and being satisfied with life were positively associated with PSA testing (Table 3). Men who had quit smoking were more likely to report having a PSA test than were nonsmoking men. Men who were divorced, widowed, or separated; rarely or never had emotional support; or had less than a high school education were less likely to report having the test than men who were married or living with partner, always had emotional support, or had more than a high school education, respectively. Veteran status and having had a stroke were no longer significantly associated with PSA testing after adjusting for all the selected variables simultaneously.

## Discussion

We found that 60% (approximately 2.7 million) of US men aged 76 or older with no history of prostate cancer reported having had a PSA test in the past year. Our findings are supported by several previous studies. Among men aged 50 or older, 57% had a PSA screening test in 2000, and the highest rate of screening (69%) was in men aged 70 to 79 (6). Data from the 2000 National Health Interview Survey (NHIS) showed that the PSA screening rate was 33% among men aged 75 or older, which accounted for approximately two-thirds of PSA tests (10). The remaining one-third of PSA tests was used for diagnostic purposes (ordered in the workup of symptoms, rather than screening of asymptomatic men) and was not included in the calculation of screening rates. We infer that the total PSA testing rate should be approximately 50%. In 2006, an analysis of linked data from the US Department of Veterans Affairs and Medicare claims found that approximately 56% of men aged 70 or older and 36% of men aged 85 or older were screened in 2003 (11). In 2009, the average rate of PSA testing in the past year was approximately 75% among men aged 75 or older (12). Data in this study came from medical chart audits of patients in 46 community-based family medicine practices in 2 northeastern networks. The estimated PSA testing rate in this study was similar to self-reported test rates generated from data of BRFSS and NHIS.

Our analyses showed certain sociodemographic and psychosocial factors and health care access were significantly associated with receipt of a PSA test. To our knowledge, potential factors associated with PSA testing have not been well characterized for men aged 75 or older. In 2003, researchers found that higher education levels and more physician visits were significantly associated with having a PSA test (10). We confirmed these findings with 2006 BRFSS data. Additionally, our analyses suggested race/ethnicity was not associated with PSA testing. We also found that men who reported being satisfied with life, always having emotional support, being married or having a partner, or having health insurance were more likely to report having a PSA test. In a quality-of-life study, higher socioeconomic status, good health, and good social relationships were consistently associated with higher life satisfaction, and emotional support had the strongest association (13). Life satisfaction and emotional support are potent predictors of well-being, an integral part of health (13). People who perceived that they were in good health were more likely to believe that they would benefit from cancer screening (14). With a better understanding of patients' sociodemographic and psychosocial characteristics, physicians may be able to employ more appropriate and effective individualized strategies to frame discussions about PSA screening, such as formulating conversations with patients on the basis of their health behaviors or incorporating spouses or partners into these discussions.

Although no major professional associations have recommended PSA screening in elderly men, high prevalences of PSA screening have been reported (6-9,11,15). PSA screening and subsequent biopsy and treatment can lead to psychological and physical harm and additional medical cost (16,17). The difficulty in estimating patients' life expectancy and the convenience and low cost of PSA testing may contribute to the overuse of PSA tests in the elderly population (10,14). In addition, misaligned financial incentives for physician practices may also partially account for this problem; physicians with a laboratory on site are more likely to order a PSA test (18). Studies have suggested that a physician's advice is a major determinant in a man's decision to have a PSA test (19,20). The new USPSTF prostate cancer guidelines should prompt clinicians to discuss the potential implications of screening for prostate cancer before ordering a PSA test for men aged 75 or older.

Our study findings are generalizable because they were generated from a nationwide population-based sampling survey. However, this study is subject to several limitations. First, the BRFSS data were based on self-report and are thus subject to recall bias. Self-reports of screening behavior overestimate the extent of actual screening (6). However, the PSA testing rate reported in our study did not exceed the rate calculated from medical chart data in 46 community-based primary care settings (12). Second, BRFSS questionnaires did not ask if people had symptoms of prostate cancer when their PSA tests were done. We could not distinguish between tests for screening (testing an asymptomatic person) and tests that help physicians make diagnoses (testing a symptomatic person). Thus, some of the PSA tests reported in our study could have been used to assist in diagnosing disease. However, a cohort study of more than 500,000 veterans aged 70 or older who had no history of prostate cancer, elevated PSA, or prostate cancer symptoms found a PSA screening rate of 56% (11). The distinction between a screening test and a diagnostic test can be difficult to make. Studies suggest that men who have lower urinary tract symptoms derived from benign prostatic hyperplasia — a common prostate disease in older men — are not at higher risk for prostate cancer, aside from the risk conferred by their age (21). Screening for prostate cancer could be defined as testing in men who do and do not have lower urinary tract symptoms (21). To be conservative, we explained our rates as the use of PSA test, instead of screening test. In an analysis of data from NHIS, 86% and 77% of the PSA tests in 1999 were used for screening among men aged 65 to 79 and 80 or older, respectively (22). Therefore, most men in our study were likely to have been asymptomatic at the time the PSA test was done. Finally, because of limited sample size, we could not examine PSA testing at state or local levels. A geographic analysis study using multiple-year BRFSS data is warranted.

In conclusion, the PSA test has been commonly used among US men aged 75 or older without prostate cancer, which might result in unnecessary physical and psychological harm and economic cost in this age group. Physicians who have previously recommended prostate cancer screening for elderly patients should consider the new USPSTF screening recommendation. Our study may help physicians and public health professionals better understand the sociodemographic and psychosocial backgrounds of these men and lay groundwork to evaluate acceptance and future implementation of the new USPSTF screening guidelines. Future studies to examine the possible change in PSA test use in this population may provide insight into the acceptability of discontinuing PSA testing in men aged 75 or older.

## Figures and Tables

**Table 1:** PSA Testing Among Men Aged 76 Years or Older With No History of Prostate Cancer, BRFSS, 2006

PSA Test	%^a^		

	All Men (N = 9,033)	Age, y	

		76-80 (n = 4,858)	≥81 (n = 4,175)
In the past year	60	63	56
1-2 Years ago	11	12	10
2-3 Years ago	4	4	4
3-5 Years ago	3	3	4
≥5 Years ago	4	2	5
Never	18	16	21

Abbreviations: PSA, prostate-specific antigen; BRFSS, Behavioral Risk Factor Surveillance System.

a Percentage population estimates adjusted for BRFSS sampling design.

**Table 2:** PSA Testing in the Past Year Among Men Aged 76 Years or Older With No History of Prostate Cancer, BRFSS, 2006

**Characteristic**	No. of Men^a^	% With PSA Test in Past Year^b^	*P* Value
**Age, y**			
76-80	4,858	63	Reference
≥81	4,175	56	<.001
**Race/ethnicity**			
Non-Hispanic white	7,817	61	Reference
Non-Hispanic black	357	54	.18
Non-Hispanic Asian or Pacific Islander	109	43	.15
Hispanic	343	63	.80
Non-Hispanic other	241	48	.42
**Education**			
More than a high school diploma	4,512	64	Reference
High school diploma	2,784	58	.01
Less than a high school diploma	1,705	52	<.001
**Marital status**			
Married or living with a partner	5,130	64	Reference
Divorced/widowed/separated	3,532	52	<.001
Never married	358	49	.004
**Health insurance**			
No	231	34	Reference
Yes	8,779	61	<.001
**Medical cost concern**			
No	8,668	60	Reference
Yes	322	57	.55
**Routine checkup in the past year**			
No	1,274	33	Reference
Yes	7,634	64	<.001
**Veteran**			
No	2,175	56	Reference
Yes	6,847	62	.02
**Smoking status**			
Nonsmoker	3,173	57	Reference
Current smoker	497	57	.96
Former smoker	5,305	62	.02
**Life satisfaction**			
Dissatisfied	326	41	Reference
Satisfied	8,509	61	<.001
**Emotional support**			
Always	5,748	63	Reference
Sometimes	810	53	.01
Rarely/none	1,802	52	<.001
**Ever had diabetes**			
No	7,271	60	Reference
Yes	1,749	59	.56
**Ever had heart attack**			
No	6,878	61	Reference
Yes	2,057	59	.55
**Ever had coronary heart disease**			
No	6,922	59	Reference
Yes	1,864	65	.02
**Ever had stroke**			
No	7,938	61	Reference
Yes	1,042	55	.04

Abbreviations: PSA, prostate-specific antigen; BRFSS, Behavioral Risk Factor Surveillance System.

a Number may differ from total because of "don't know," refused, or missing responses.

b Percentage population estimates adjusted for BRFSS sampling design.

**Table 3:** Associations Between PSA Testing and Various Characteristics Among Men Aged 76 Years or Older With No History of Prostate Cancer, BRFSS, 2006

**Characteristic**	OR (95% CI)
**Age, y**	
76-80	1.00 [Reference]
≥81	0.78 (0.66-0.92)^a^
**Race/ethnicity**	
Non-Hispanic white	1.00 [Reference]
Non-Hispanic black	0.92 (0.60-1.43)
Non-Hispanic Asian or Pacific Islander	0.43 (0.14-1.27)
Hispanic	1.28 (0.78-2.10)
Non-Hispanic other	0.61 (0.36-1.05)
**Education**	
More than a high school diploma	1.00 [Reference]
High school diploma	0.85 (0.71-1.02)
Less than a high school diploma	0.70 (0.55-0.90)^a^
**Marital status**	
Married or living together	1.00 [Reference]
Divorced/widowed/separated	0.71 (0.60-0.84)^a^
Never married	0.69 (0.46-1.04)
**Health insurance**	
No	1.00 [Reference]
Yes	2.63 (1.44-4.71)^a^
**Medical cost concern**	
No	1.00 [Reference]
Yes	1.27 (0.78-2.07)
**Routine checkup in the past year**	
No	1.00 [Reference]
Yes	3.73 (2.89-4.82)^a^
**Veteran**	
No	1.00 [Reference]
Yes	1.13 (0.92-1.40)
**Smoking status**	
Nonsmoker	1.00 [Reference]
Current smoker	1.09 (0.70-1.69)
Former smoker	1.22 (1.02-1.45)^a^
**Life satisfaction**	
Dissatisfied	1.00 [Reference]
Satisfied	1.77 (1.09-2.87)^a^
**Emotional support**	
Always	1.00 [Reference]
Sometimes	0.87 (0.63-1.20)
Rarely/none	0.77 (0.63-0.96)^a^
**Ever had diabetes**	
No	1.00 [Reference]
Yes	0.86 (0.69-1.08)
**Ever had heart attack**	
No	1.00 [Reference]
Yes	0.83 (0.70-1.06)
**Ever had coronary heart disease**	
No	1.00 [Reference]
Yes	1.27 (1.00-1.60)^a^
**Ever had stroke**	
No	1.00 [Reference]
Yes	0.84 (0.65-1.07)

Abbreviations: PSA, prostate-specific antigen; BRFSS, Behavioral Risk Factor Surveillance System; OR, odds ratio; CI, confidence interval.

a Significant at *P* < .05.

## Appendix: Behavioral Risk Factor Surveillance System Telephone Survey Questions Assessing Use of Prostate-Specific Antigen Test and Diagnosis of Prostate Cancer Among US Men Aged 76 Years or Older

1. A prostate-specific antigen test, also called a PSA test, is a blood test used to check men for prostate cancer. Have you ever had a PSA test?

- Yes
- No
- Do not know/not sure
- Refused

2. How long has it been since you had your last PSA test?

- Anytime less than 12 months ago
- One year but less than 2 years
- Two years but less than 3 years
- Three years but less than 5 years
- Five or more than 5 years

3. Have you ever been told by a doctor, nurse, or other health professional that you had prostate cancer?

- Yes
- No
- Do not know/not sure
- Refused
